# Landau-Zener-Stückelberg interference in coherent charge oscillations of a one-electron double quantum dot

**DOI:** 10.1038/s41598-018-23468-2

**Published:** 2018-04-03

**Authors:** Takeshi Ota, Kenichi Hitachi, Koji Muraki

**Affiliations:** NTT Basic Research Laboratories, NTT Corporation, 3-1 Morinosato-Wakamiya, Atsugi, 243-0198 Japan

## Abstract

Landau-Zener (LZ) transition has received renewed interest as an alternative approach to control single-qubit states. An LZ transition occurs when a system passes through an avoided crossing that arises from quantum mechanical coupling of two levels, taking the system to a coherent superposition of the two states. Then, multiple LZ transitions induce interference known as Landau-Zener-Stückelberg (LZS) interference whose amplitude strongly depends on the velocity or adiabaticity of the passage. Here, we study the roles of LZ transitions and LZS interference in coherent charge oscillations of a one-electron semiconductor double quantum dot by time-domain experiments using standard rectangular voltage pulses. By employing density matrix simulations, we show that, in the standard setup using rectangular pulses, even a small distortion of the pulse can give rise to LZ transitions and hence LZS interference, which significantly enhances the measured oscillation amplitude. We further show experimentally that the nature of the coherent charge oscillations changes from Rabi-type to LZS oscillations with increasing pulse distortion. Our results thus demonstrate that it is essential to take into account LZS interference for both precise control of charge qubits and correct interpretation of measurement results.

## Introduction

A quantum two-level system forms the basis for studying and controlling the dynamics of quantum coherent phenomena in terms of quantum information physics, and is a building block of a quantum bit, or a qubit^1^. The standard architecture of qubit operation is based on the premise that any single-qubit gate can be constructed from a sequence of pulses that change the system’s effective Hamiltonian non-adiabatically. The evolution of the qubit state is described by successive rotations of the state vector on the Bloch sphere, and the state is read out by projecting it on an appropriately chosen measurement basis, typically Pauli matrix σ_*z*_. In the solid state, various quantum two-level systems have been implemented^2–9^, and quantum coherent oscillations driven by pulsing the system have been reported as a demonstration of successful qubit operations.

Recently, Landau-Zener (LZ) transitions^10–12^ have received renewed interest as an alternative approach to control single-qubit states^13^. An LZ transition occurs when a system passes through an avoided crossing that arises from quantum mechanical coupling of two levels. Except for the fully adiabatic or non-adiabatic limits, an LZ transition takes the system to a coherent superposition of the two states with the relative amplitudes determined by the avoided crossing gap and the velocity, or adiabaticity, of the passage. By appropriately tuning the velocity, a single passage can create a coherent superposition with equal weights, serving as a 50:50 beam splitter for the incoming state. Successively sweeping back and forth through the avoided crossing thus induces multiple LZ transitions, resulting in the interference between the outgoing states. This phenomenon, known as Landau-Zener-Stückelberg (LZS) interference^10–12^, has been proposed as a method to control qubits without the need for sharp pulses^13^, and observed in various quantum two-level systems in the solid state based on Josephson junctions^14,15^, coupled donor states^16^, and semiconductor double quantum dots (DQDs)^7,17–20^.

In ref.^20^, coherent oscillations arising from LZS interference (hereafter LZS oscillations) between singlet and triplet spin states in a DQD were demonstrated by sweeping through an avoided crossing induced by nuclear spins, where the long time scale (of the order of 10 ns) set by the small gap facilitated the precise velocity control. On the other hand, in semiconductor charge qubits^5–7^, due to the large avoided crossing gap, typically 10 μeV, the relevant time scale is in the range of several tens of picoseconds, where accurate velocity control becomes a greater challenge. Consequently, LZS interference in charge qubits has been studied in the context of photon-assisted interdot tunneling under continuous microwave irradiation^16,17^. Time-domain observation of LZS interference has been reported only recently in ref.^18^, where the velocity was controlled by varying the amplitude of a fixed-length short (150 ps) pulse or shaping a rectangular pulse with a low-pass filter.

In this paper, we study the roles of LZ transitions and LZS interference in coherent charge oscillations of a one-electron semiconductor DQD by time-domain experiments using standard rectangular voltage pulses. By employing density matrix simulations, we show that, in the standard setup using rectangular pulses, even a small distortion of the pulse can give rise to LZ transitions and hence LZS interference, which significantly enhances the measured oscillation amplitude. We further show experimentally that the nature of the coherent charge oscillations changes from Rabi-type to LZS oscillations with increasing pulse distortion. Our results thus demonstrate that it is essential to take into account LZS interference for both precise control of charge qubits and correct interpretation of measurement results.

## Landau-Zener-Stückelberg Interference

**Figure 1:**
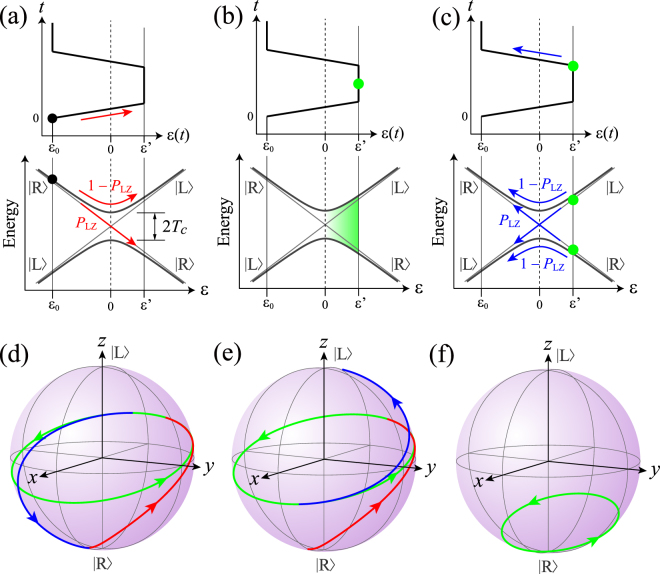
(**a**–**c**) Charge dynamics of one-electron DQD driven by a trapezoidal pulse. Panels (a–c) represent three steps: (**a**) first LZ transition, (**b**) phase accumulation, and (**c**) second LZ transition. State evolution is shown by circles and arrows in the upper and lower diagrams displaying detuning vs time and energy vs detuning. **(d**,**e)** Corresponding state evolution on the Bloch sphere simulated for slightly different pulse lengths leading to (**d**) destructive and (**e**) constructive LZS interference. Trajectories shown in red, green, and blue represent state evolution in steps (**a**), (**b**), and (**c**), respectively. Parameters for these simulations were chosen to yield *P*_LZ_ close to 0.5 (*T*_C_ = 11 μeV, ε_0_ = −60 μeV, Δε = 120 μeV, and pulse rise/fall time of 81 ps). (**f**) State evolution for a non-adiabatic rectangular pulse with the same height.

We consider a one-electron state in a DQD and take the basis to be |L〉 and |R〉, in which the electron occupies the left and right dot, respectively. Using Pauli matrices σ_*x*_ and σ_*z*_, the Hamiltonian of the system can be written as^5^

$${H}=\frac{1}{2}{\rm{\varepsilon }}({t})\,{{\rm{\sigma }}}_{{z}}+{T}{}_{{C}}{\rm{\sigma }}_{{x}},$$

where ε(*t*) = *E*_L_(*t*) − *E*_R_(*t*) is the detuning between the energy levels in the left and right dots, and *T*_C_ is the interdot tunnel coupling energy. Here we assume without loss of generality that the initial state is prepared in |R〉 with ε set to a large negative value ε_0_ at *t* < 0 [Fig. 1(a)] (see Methods). In the standard qubit operations, coherent Rabi-type oscillations between |R〉 and |L〉 are triggered by switching ε non-adiabatically to a value ε′ close to the resonance ε = 0, and then the state is projected onto the measurement basis for readout by switching ε back to ε_0_. The important observation here is that the qubit manipulation for ε′ > 0 inevitably involves passages through the avoided crossing at ε = 0. Therefore, if ε changes at a finite velocity *v* ≡ |dε/d*t*|, each passage will induce an LZ transition with the asymptotic probability ${P}_{{\rm{LZ}}}=\exp (-2\,\pi \delta )$, where ${\rm{\delta }}={T}_{C}^{2}/\hslash v$ ($\hslash$ is Planck’s constant divided by 2π)^20^, which then gives rise to LZS interference when ε is set back to ε_0_ for the readout [Fig. 1(c)]. Note that δ → 0(∞) corresponds to the non-adiabatic (adiabatic) limit, for which *P*_LZ_ → 1(0).

The impacts of LZ transitions and LZS interference on the qubit manipulation can be better understood by using the Bloch sphere representation. It is known that the dynamics at an LZ transition can be expressed by a unitary operation ${U}_{{\rm{LZ}}}={R}_{z}(-{\tilde{{\rm{\phi }}}}_{{\rm{S}}}){R}_{x}({\theta }_{{\rm{LZ}}}){R}_{z}(-{\tilde{{\rm{\phi }}}}_{{\rm{S}}})$ that represents successive rotations of the Bloch vector for the incoming state^15^. Here, ${R}_{x}({\theta }_{{\rm{LZ}}})=\exp (-{\rm{i}}{\theta }_{{\rm{LZ}}}{\sigma }_{x}/2)$ and ${R}_{z}(-{\tilde{{\rm{\phi }}}}_{{\rm{S}}})=\exp ({\rm{i}}{\tilde{{\rm{\phi }}}}_{{\rm{S}}}{\sigma }_{z}/2)$ describe rotations around the *x*- and *z*-axes by angles θ_LZ_ and $-{\tilde{{\rm{\phi }}}}_{{\rm{S}}}$, respectively. θ_LZ_ and ${\tilde{{\rm{\phi }}}}_{{\rm{S}}}$ are given by ${\theta }_{{\rm{LZ}}}=2{\sin }^{-1}\sqrt{{P}_{{\rm{LZ}}}}$ and ${\tilde{{\rm{\phi }}}}_{{\rm{S}}}=\text{arg}[\Gamma (1-{\rm{i}}\delta )+\delta (ln\delta -1)]-\pi /4$, where Γ is the Gamma function^15^. Using these representations, LZS interference can be expressed as *U*_LZ_*R*_*z*_(φ_p_)*U*_LZ_, where ${{\rm{\phi }}}_{{\rm{p}}}={\hslash }^{-1}\int [{E}_{{\rm{L}}}(t)-{E}_{{\rm{R}}}(t)]\,dt$ is the phase accumulated during the *z* rotation between the first and second LZ transitions. In this idealized model, the *z* component of the Bloch vector changes only via the two *x* rotations, but their effects can be constructive or destructive^21^, depending on the *z* rotation angle—that is, the phase accumulated—between them. This phase-dependent action of the two *x* rotations is the essence of LZS interference.

To illustrate these, we computed the trajectories of the Bloch vector driven by trapezoidal pulses with slightly different lengths [Fig. 1(d) and (e)]. The parameters *T*_C_ and *v* were chosen in such a way that *P*_LZ_ ∼ 0.5. The use of a trapezoidal pulse shape allows us to decompose the dynamics into three steps: the first LZ transition [Fig. 1(a)], phase accumulation [Fig. 1(b)], and the second LZ transition [Fig. 1(c)]. The trajectories in the time domains corresponding to the three steps are shown in red, green, and blue, respectively, in Fig. 1(d) and (e). The simulations confirm that both the first and second LZ transitions can be approximately viewed as combinations of *x* and *z* rotations. In these simulations, the pulse lengths were chosen for the effects of the two *x* rotations to be the most destructive [Fig. 1(d)] or the most constructive [Fig. 1(e)]. Consequently, after the second LZ transition the direction of the Bloch vector spans almost the full range from the south to north poles. Note that this is possible because we set *P*_LZ_ ∼ 0.5 (i.e., θ_LZ_ ∼ π/2), which makes each *x* rotation serve as a π/2 pulse. It is instructive to compare these results with the case for a non-adiabatic rectangular pulse with the same height [Fig. 1(f)]. Since θ_LZ_ = 0 in this case, the trajectory stays near the south pole. The amplitude of this Rabi-type oscillation determined by ε′ and *T*_C_ alone is much lower than 1 for an off-resonant pulse as shown in Fig. 1(f). These observations indicate that the finite adiabaticity of the pulse can lead to LZS interference, where the amplitude of the measured oscillations may significantly exceed that expected for an ideal non-adiabatic pulse.

## Measurements using rectangular pulses

**Figure 2:**
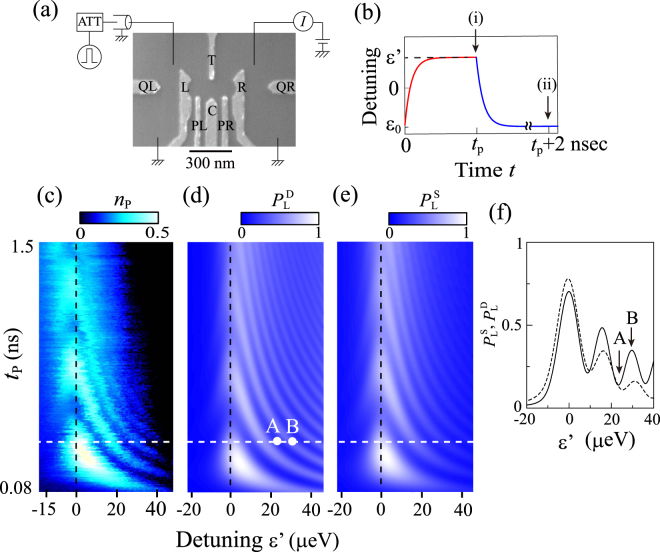
**(a)** Schematic illustration of the experimental setup. High-frequency voltage pulses are applied to the drain electrode. **(b)** Schematic of the pulse shape used for the simulations in (**d**) and (**e**). Arrows (i) and (ii) indicate the points of state projection in the single- and double-passage models, respectively. **(c)** Plot of the average number of pulse-induced tunneling electrons, *n*_p_, as a function of pulse duration *t*_p_ and detuning ε′ during the pulse-on period. Larger values of *n*_p_ indicate higher probability of finding the state in |L〉. Measurements were done with Δε = 65 μeV and *f*_rep_ = 50 MHz in the strong dot-lead coupling regime. Gate voltages *V*_T_ and *V*_C_ applied to gates T and C in Fig. 2(a) were −0.74 and −0.866 V, respectively. **(d**,**e)** Density-matrix calculations showing the probabilities of finding the state in |L〉 obtained in the (**d**) double-passage and (**e**) single-passage models (*P*_L_^D^ and *P*_L_^S^) by taking the state projection at *t* = *t*_p_ + 2 ns and *t*_p_, respectively. Parameters used are *t*_d_ = 30 ps, Δε = 65 μeV, and Γ = 1 ns^−1^. **(f)** Line cuts of the plots in (**d**) and (**e**) taken along the white dashed line at *t*_p_ = 0.32 ns, shown in solid and dashed line, respectively. The points A and B correspond to those in (**d**).

Figure 2(a) schematically shows the device structure and experimental setup. A DQD is formed in a two-dimensional electron gas at the interface of a GaAs/AlGaAs heterojunction by applying negative voltages to the surface Schottky metal gates. We use gate voltages *V*_L_ and *V*_R_ to vary the electron number in the DQD and *V*_C_ to tune the interdot tunnel coupling energy *T*_C_. We focus on the one-electron regime of the DQD. High-frequency voltage pulses are applied to the drain electrode of the DQD, which provides fast control of the dot energy levels and hence the detuning ε(*t*). Experiments were performed at a lattice temperature of 20 mK. A magnetic field of 0.2 T was applied perpendicular to the sample to eliminate unwanted level degeneracy.

First, we present results for the standard single-qubit operation exhibiting Rabi-type oscillations. For this experiment, the gate voltages were tuned to allow for strong dot-lead coupling so that the charge state in the DQD could be readout by measuring the current flowing through the DQD as in ref.^5^ (for details, see Methods). Rectangular voltage pulses with amplitude corresponding to a change in the detuning of Δε ≡ ε′ − ε_0_ = 65 μeV were applied to the drain electrode at repetition frequency *f*_rep_ = 50 MHz. Figure 2(c) shows the average number of pulse-induced tunneling electrons, *n*_p_ = *I*_p_/*ef*_rep_, plotted as a function of pulse duration *t*_p_ and detuning ε′ during the pulse-on period. The data show behavior characteristic of Rabi-type oscillations. From the oscillation frequency at the resonance (ε′ = 0), *T*_C_ is estimated to be 4.25 μeV. We focus on the asymmetry with respect to ε′ = 0; clearly, the oscillations are much more pronounced at ε′ > 0. Similar asymmetry has been seen not only in semiconductor charge qubits^5,6^ but also in superconducting charge qubits^8^. Recently, effects of LZS interference on coherent charge oscillations were theoretically studied in ref.^22^, where it was argued that the asymmetry was a clear signature of coherent LZS oscillations. However, as will be shown below, the asymmetry can arise from the finite slope of the rising edge of the pulse alone, i.e., without LZS interference. To elucidate the effects of LZS interference in Rabi-type oscillations, below we examine the state evolution during the falling time as well as the rising time of the pulse.

We simulate the time evolution of the system by solving the master equation for a reduced density matrix *ρ* with the effects of decoherence taken into account in the Lindblad formalism:

$$\frac{{\rm{d}}\rho }{{\rm{d}}t}=\frac{1}{{\rm{i}}\hslash }[H,\rho ]+(L\rho \,{L}^{+}-\frac{1}{2}{L}^{+}L\rho -\frac{1}{2}\rho \,{L}^{+}L).$$

Here, *L* is the Lindblad operator defined as $L=\sqrt{{\rm{\Gamma }}}(|{\rm{ES}}\rangle \langle {\rm{ES}}|-|{\rm{GS}}\rangle \langle {\rm{GS}}|)$, Γ (=1/*T*_2_) is the decoherence rate of the charge state, and |GS〉 (|ES〉) is the instantaneous ground (excited) state of the two-level system. We model the pulse distortion by taking the convolution of a rectangular pulse with an exponential function ${e}^{-t/{t}_{d}}$ [Fig. 2(b)], where *t*_d_ represents the finite response time of the dot potential to the applied pulse. To distinguish the effects of LZ transitions and LZS interference, we compare the probabilities of finding the state in |*L*〉 for state projection at different times, (i) *t* = *t*_p_ and (ii) *t*_p_ + 2 ns, i.e., just before and sometime after the pulse is turned off [see arrows in Fig. 2(b)]. Note that LZS interference is involved only in the latter. In the following, we refer to (i) and (ii) as “single-passage” and “double-passage” models, respectively.

Figure 2(d) and (e) show the results for the double- and single-passage models, respectively, calculated for *t*_d_ = 30 ps, *T*_C_ = 4.25 μeV, Γ = 1 ns^−1^, and Δε = 65 μeV. These parameters were chosen to fit the coherent oscillations at ε′ = 0 (for details, see Methods). The figures plot the probability of finding the state in |*L*〉, which we denote by *P*_L_^S^ (*P*_L_^D^) for the single (double) passage model. The two models yield similar results—both reproduce the observed behavior well, including the asymmetry with respect to ε′ = 0. (Although not shown, the asymmetry is absent for a perfectly rectangular pulse with *t*_d_ = 0.) It should be noted, however, that the contrast between ε′ > 0 and ε′ < 0 is much more pronounced in *P*_L_^D^. It is particularly noteworthy that for *P*_L_^D^ pronounced oscillations persist up to large ε′. Figure 2(f) compares the line cuts of *P*_L_^S^ and *P*_L_^D^ at *t*_p_ = 0.32 ns. In both traces, the oscillation amplitude decreases with increasing ε′, but much more slowly in *P*_L_^D^ [solid trace in Fig. 2(f)]. This is because LZS interference not only enhances the oscillation amplitude but also renders it less dependent on ε′. Note that in the experiment the amplitude decays faster with increasing ε′ [Fig. 2(c)]. This is because in reality Γ increases with |ε′|, due to additional decoherence induced by background charge fluctuations^5,6^.

**Figure 3:**
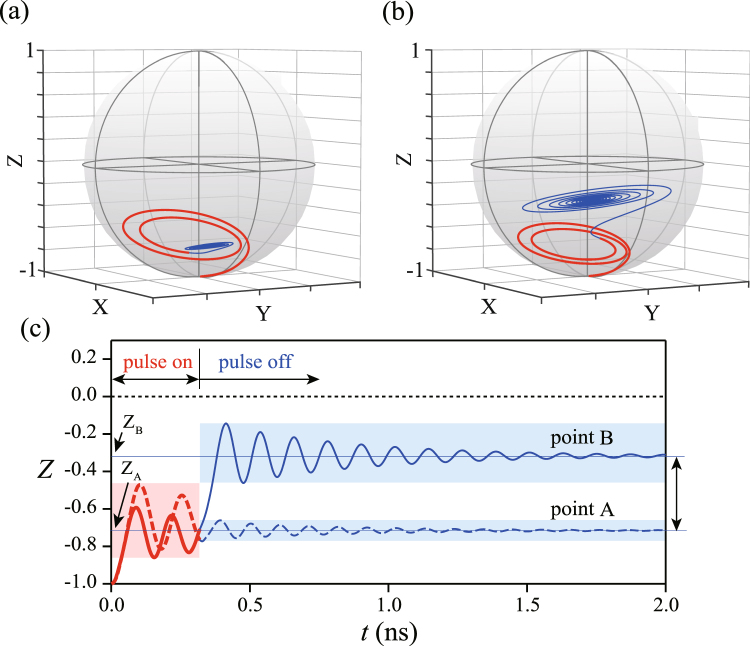
(**a**,**b**) Trajectories of the Bloch vector representing the time evolution at two slightly different ε′ values corresponding to the (**a**) minimum and (**b**) maximum in *P*_L_^D^ for *t*_p_ = 0.32 ns [points A and B in Fig. 2(d) and (f), respectively]. Trajectories shown in red and blue represent time evolution during the “pulse-on” and “pulse-off” periods, respectively. (**c**) Projected *z* component of the trajectories shown in (**a**) [(b)]. “Pulse-on” and “pulse-off” periods are shown in red and blue, respectively. Z_A_ and Z_B_ represent the *z* component averaged over time in the pulse-off period.

Figure 3(a) and (b) compare the trajectories of the Bloch vector for pulses with the same *t*_p_ (=0.32 ns) but at slightly different ε′ values corresponding to the minimum and maximum in *P*_L_^D^ [marked “A” and “B” in Fig. 2(d) and (f)]. The trajectories for *t* < *t*_p_ (*t* > *t*_p_) are shown in red (blue). The system follows similar trajectories for *t* < *t*_p_: the state initially located at the south pole (|*R*〉) is moved toward the equator by the *x* rotation that accompanies the first LZ transition before exhibiting a *z* rotation driven by the off-resonant pulse. In contrast, the trajectories for *t* > *t*_p_ are entirely different: in Fig. 3(a) the state remains near the south pole, whereas in Fig. 3(b) it shifts further toward the equator, where it makes a greater circle. The *z* projections of these trajectories are plotted in Fig. 3(c) as a function of *t*. Note that in the double-passage model the measured signal corresponds to the time average of the *z* component in the pulse-off period, labeled *Z*_A_ and *Z*_B_ in Fig. 3(c). As seen in Fig. 3(c), the high contrast between the maxima (*Z*_B_) and minima (*Z*_A_) of the oscillations that is to be measured in the experiment arises from the post-pulse dynamics shown in blue. This indicates that, although the overall features of the oscillations in Fig. 2(d) share those of Rabi-type oscillations, the dominant contributor to the oscillation amplitude is LZS interference. The existence of such post-pulse dynamics in a semiconductor charge qubit is corroborated by the observation of Ramsey fringes in ref.^23^. Note that *t*_p_ is fixed in the present case, so the interference phase is tuned via ε′, which determines the *z* rotation frequency.

## Measurements using largely distorted pulses

For the parameters used above, *P*_LZ_ values for the rising and falling edges of the pulse are in the range of 0.25 to 0.3. The signature of LZS interference is expected to be further strengthened when *P*_LZ_ becomes closer to 0.5. Since high-frequency voltage pulses are applied to the drain electrode of the DQD in our experimental setup, the actual pulse shape experienced by the electron in the DQD could be modified by the capacitive component around the lead to the DQD. As we show below, the pulse shape becomes largely distorted when the isolation between the dot and lead is increased by applying a larger negative gate voltage. In the following measurement, the dot-lead coupling was set in a weak coupling regime, where the current flowing through the DQD is no longer measurable. The charge state of the DQD was therefore detected by measuring the current *I*_QPC_ that flows through the QPC charge sensor attached nearby the DQD.

**Figure 4:**
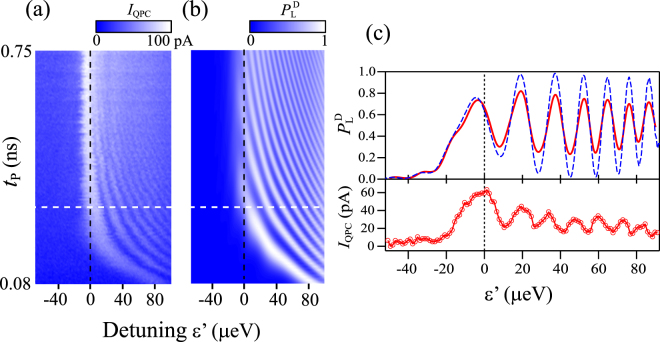
(**a**) Plot of the pulse-induced QPC current *I*_QPC_ as a function of pulse duration *t*_p_ and detuning ε′ during the pulse-on period. Measurements were done in the weak dot-lead coupling regime with Δε = 145 μeV, *f*_rep_ = 25 MHz, *V*_T_ = −1 V, and *V*_C_ = −0.7 V. **(b)** Density-matrix calculation showing the probability of finding the state in |L〉, *P*_L_^D^, obtained in the double passage model for *t*_d_ = 100 ps, Δε = 145 μeV, and Γ = 1 ns^−1^. **(c)** Line cuts of experimental data in (**a**) (circles in the lower panel) and simulation in (**b**) (solid line in the upper panel) taken at *t*_p_ = 0.3 ns along the white dashed line in (**a**) and (**b**). The dashed line in the upper panel represents simulation for Γ = 0. Additional fine interference fringes that appear for Γ = 0 were removed by numerically averaging the data along the ε′ axis.

Figure 4(a) shows the pulse-induced QPC current *I*_QPC_ as a function of pulse duration *t*_p_ and detuning ε′ (Δε = 145 μeV and *f*_rep_ = 250 MHz) (for details, see Methods). We find that the gross features of the data are largely different from those in Fig. 2(c). Now the coherent oscillations at ε′ = 0 are barely visible. Oscillatory behavior is seen only at ε′ > 0, i.e., only when the state passes through the avoided crossing. Furthermore, the oscillations maintain high visibility over a wider range of ε′ (>0). Consequently, the data possess the general features of LZS oscillations^18,20^. Figure 4(b) shows the probability of finding the state in |*L*〉, *P*_L_^D^, as a function of ε′ and *t*_p_, calculated in the double-passage model with the state projection at *t* = *t*_p_ + 2 ns. With appropriately chosen parameters (*t*_d_ = 100 ps, *T*_C_ = 7.5 μeV, and Γ = 1 ns^−1^), the simulation well reproduces the experimentally observed behavior. Here, the long *t*_d_ of 100 ps is essential to the LZS oscillation-like behavior. The corresponding *P*_LZ_ values are in the range of 0.45 to 0.5. Because the *P*_LZ_ values become closer to 0.5, LZS interference plays an even more important role in the measured coherent oscillations. To highlight the signature of LZS interference, line cuts of the plots in Fig. 4(a) and (b) at *t*_p_ = 0.3 ns are shown in Fig. 4(c). As the simulation (solid line in the upper panel) shows, the ε′ dependence of the oscillation amplitude is much weaker than in Fig. 2(f). The weak ε′ dependence is evident also in the experiment [circles in the lower panel of Fig. 4(c)], though the oscillation amplitude is much reduced.

## Discussion

When the state evolution is well described by the actions of successive *x* and *z* rotations, the probability of finding the state in |L〉 after the pulse (with the initial state being |R〉) can be expressed as

$$P=(1-{P}_{1}){P}_{2}+(1-{P}_{2}){P}_{1}-2\sqrt{{P}_{1}{P}_{2}(1-{P}_{1})(1-{P}_{2})}\,\cos \,\,{{\rm{\phi }}}_{{\rm{p}}}$$

where *P*_1_ and *P*_2_ are the asymptotic LZ transition probabilities at the rising and falling edges of the pulse, respectively^24^. Owing to the phase-dependent term, *P* oscillates as a function of ε′ and *t*_p_, with the maxima (minima) indicating that the interference between the outgoing states is the most constructive (destructive)^21^. In our experiment, *P*_1_ and *P*_2_ are not equal and both vary with the detuning as a result of the non-trapezoidal pulse shape. The amplitude of the interference fringe $F=2\sqrt{{P}_{1}{P}_{2}(1-{P}_{1})(1-{P}_{2})}$ reaches a maximum value *F* = 0.5 for *P*_1_ = *P*_2_ = 0.5, where *P* oscillates between 0 and 1. In our experiment, the *F* value estimated from the *t*_d_ of 100 ps (30 ps) that provides a good fit to the experiment in the weak (strong) dot-lead coupling regime is about 0.5 (less than 0.3). As shown by the dashed line in the upper panel of Fig. 4(c), the density-matrix calculation for *t*_d_ = 100 ps without dephasing (i.e., Γ = 0) shows oscillations between nearly 0 and 1. This confirms that the simple analysis based on the asymptotic probabilities *P*_1_ and *P*_2_ captures the essence of the physics.

In summary, we studied the impact of LZS interference on coherent charge oscillations in a one-electron DQD. By numerical simulation, we found a significant enhancement of the oscillation amplitude due to LZS interference when the system traverses the avoided crossing. Our results demonstrate that LZS interference is inherent to charge qubits. This indicates that appropriate tuning of the pulse shape and an analysis including after-pulse dynamics are essential for the precise control of charge qubits and correct interpretation of the measurement results.

## Methods

### Experimental procedure

The device was fabricated from a GaAs/AlGaAs heterostructure wafer with electron density of 2.3 × 10^15^ m^−2^ and mobility of 180 m^2^/Vs. The two-dimensional electron gas formed at the heterointerface is located 10 nm below the surface. Rectangular high-frequency voltage pulses with amplitude corresponding to a change in detuning Δε and duration time *t*_p_, applied to the drain electrode of the DQD, allow for a fast control of the dot energy levels. We employed two different detection schemes for reading out the charge state of the DQD, depending on the strength of the dot-lead coupling. In the strong coupling regime, we measured dot current *I*_p_ flowing from the source to drain through the DQD as in ref.^5^. In the weak coupling regime, *I*_p_ is no longer measurable, so we used a nearby quantum point contact (QPC) charge sensor set in the linear tunneling regime^6^. We employed a lock-in technique to improve the signal-to-noise ratio by chopping the pulses at 100 Hz.

**Figure 5:**
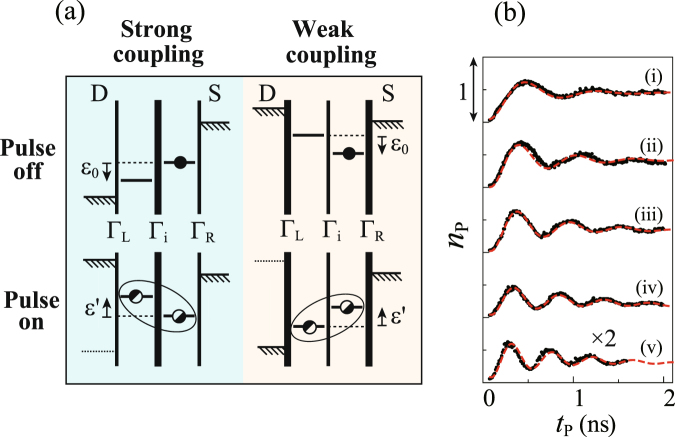
(**a**) Schematics of the DQD level alignment during the “pulse-on” and “pulse-off” periods for the strong and weak dot-lead coupling regimes. **(b**) Average number of pulse-induced tunneling electrons, *n*_p_ as a function of pulse duration *t*_p_ taken at ε′ = 0 in the strong dot-lead coupling regime for different interdot tunnel coupling strength *T*_C_. *T*_C_ was tuned by varying *V*_C_ as (i) −0.884, (ii) −0.880, (iii) −0.876, (iv) −0.872, and (v) −0.868 V, with *V*_T_ fixed at −0.74 V. The *T*_C_ values estimated from the fitting are (i) 2.6, (ii) 3.3, (iii) 3.6, (iv) 4.1, and (v) 4.6 μeV.

The energy level alignments of the DQD during the pulse on and off periods are depicted in Fig. 5(a) for the cases of strong and weak couplings. Note that the polarity of the pulse is opposite in the strong- and weak-coupling cases and, accordingly, the sign of detuning is reversed between them. We therefore use different sign definitions of detuning as ε = ±(*E*_L_ − *E*_R_) for the strong (+) and weak (−) couplings, in such a way that ε_0_ < 0 and the system passes through the avoided crossing for ε′ > 0 in both cases. This allows us to use the Hamiltonian of the same form.

As shown in Fig. 5(a), the DQD is initialized in |R〉 in both strong- and weak-coupling cases in spite of the opposite polarity of the pulse. For the strong-coupling case, this happens because of the large source-drain bias applied during the pulse-off period and the large ratio between the dot-lead coupling Г_R(L)_ and the interdot coupling Г_i_ (≪Г_R_, Г_L_)^5^. Note that during the pulse-on period the DQD is effectively isolated from the electrodes by the Coulomb blockade. After the pulse, the electron can tunnel out to the drain electrode and contribute to *I*_p_ only if the final state is |L〉. In the weak-coupling regime, the small tunneling rate Г_L_ through the left barrier prevents the electron from escaping to the drain electrode during the pulse-on period.

### Coherent charge oscillations

Figure 5(b) shows *I*_p_ vs *t*_p_ measured at ε′ = 0 in the strong dot-lead coupling regime for different interdot tunnel coupling strength *T*_C_. The oscillations at ε′ = 0, where LZ transitions are not involved, demonstrate coherent Rabi-type oscillations between |R〉 and |L〉. The oscillation frequency changes as *T*_C_ is increased by setting *V*_C_ less negative. By fitting the oscillations with a damped cosine function ${n}_{{\rm{p}}}({t}_{{\rm{p}}})=A-B\,\exp (-{t}_{{\rm{p}}}/{T}_{2})\cos ({\rm{\Omega }}{t}_{{\rm{p}}})+C{t}_{{\rm{p}}}$, *T*_C_ (≡$\hslash$Ω/2) is estimated to be 2.6 ~ 4.6 μeV. (*A*, *B*, *C*, and *T*_2_ are fit parameters.) The decoherence rate Γ (=1/*T*_2_) obtained from the fitting is ~1 ns^−1^ almost independent of *T*_C_, indicating that it is limited by decoherence due to cotunneling between dot and leads^5,6^.
